# The Relationship Between Anxiety, Stress, Depression, Traumatic Childbirth Perception, and Prenatal Attachment in Pregnant Women: A Cross‐Sectional Study

**DOI:** 10.1155/da/5941464

**Published:** 2026-05-26

**Authors:** Hüsne Yücesoy, Fatma Yildirim, Mevlüde Alpaslan Arar, Nülüfer Erbil

**Affiliations:** ^1^ Ordu University, Ordu, Türkiye, odu.edu.tr; ^2^ Department of Obstetrics and Gynecology Nursing, Faculty of Health Sciences, Hitit University, Çorum, Türkiye, hitit.edu.tr; ^3^ Samsun Provincial Health Directorate, Samsun, Türkiye; ^4^ Department of Obstetrics and Gynecologic Nursing, Faculty of Health Sciences, Ordu University, Ordu, Türkiye, odu.edu.tr

**Keywords:** anxiety, depression, pregnancy, prenatal attachment, stress, traumatic childbirth perception

## Abstract

**Aim:**

This study aims to examine the relationships between perception of traumatic childbirth, prenatal attachment, stress, anxiety, and depression in pregnant women.

**Method:**

This cross‐sectional study involved 304 pregnant women who visited the obstetrics and gynecology outpatient clinic of a hospital in Türkiye between June 2024 and June 2025. Data were collected using a Demographic Information Form, the Depression, Anxiety, Stress Scale (DASS‐21), the Traumatic Childbirth Perception Scale (TCPS), and the Prenatal Attachment Inventory (PAI). Descriptive statistical methods, Pearson correlation, and multiple linear regression analysis were used in the analysis of the data.

**Results:**

The study determined that 22% of pregnant women experienced stress, 45.1% experienced depression, and 62.8% experienced anxiety. The mean PAI score of pregnant women was found to be 40.53 ± 7.97, and the mean TCPS score was 71.64 ± 28.02 (moderate). The study found that stress, depression, and anxiety scores in pregnant women were significantly correlated positively with the TCPS score and significantly correlated negatively with the PAI score. Depression (*p* = 0.003) and prenatal attachment (*p* = 0.018) were determined to be the predictors of traumatic birth perception.

**Conclusion:**

This study determined that as women’s depression, stress, anxiety, and levels increased, their perception of traumatic childbirth increased, and their prenatal attachment levels decreased. Depression and prenatal attachment were identified as predictors of traumatic childbirth perception. Providing mental health support to pregnant women by nurses can contribute to the protection and improvement of maternal and infant health.

## 1. Inroduction

Pregnancy marks a significant transition for women, bringing about physical and psychological changes, as well as a dynamic renegotiation of their identity within the family and society [1]. Women often face psychological challenges during pregnancy, and this can negatively affect their own health, as well as the health of their fetus, in the long term [2]. Psychological health during pregnancy includes various emotions and states, such as sadness, fear, stress, depression, and anxiety [3]. Among these, depression, stress, and anxiety are the most common and often occur simultaneously [4]. The prevalence of depression in pregnant women is reported to range from 7% to 37.1% [5], and the global prevalence of anxiety is reported to be between 15% and 20% [6]. The prevalence of perceived stress is reported to range from 5.5% to 15% in developed countries and from 33% to 52.9% in developing countries [7–9].

Depression, anxiety, and stress during pregnancy are reported to be significantly associated with a woman’s perception of childbirth as traumatic [10–13]. In addition, it has been reported that a negative perception of childbirth can lead to anxiety and depression in women and negatively affect their psychosocial health [12]. The perception of traumatic childbirth, also called psychological birth trauma, is defined as a woman’s perception of childbirth as an event that will harm herself or her baby at any stage of the fertile process [14, 15]. Fear of childbirth, on the other hand, refers to various thoughts and frightening feelings related to childbirth, encompassing a spectrum ranging from minor fears (normal fear) to severe fears (fear affecting daily activities) [16]. There is no standard definition of fear of childbirth, and it generally refers to feelings of fear, anxiety, or worry related to pregnancy and childbirth [17]. Therefore, while the two concepts are related, they are not equivalent. This study adopts the framework of the perception of traumatic childbirth.

Perceptions about labor influence every stage of pregnancy and the birth experience. They can be the key to a negative memory that a woman will cherish for a lifetime, or to a wonderful childbirth experience [12]. The quality of the birth experience is related to the quality of prenatal attachment and postnatal mother‐infant bonding [18]. In recent decades, the terms “post‐partum bond” or “parent‐infant bonding” have frequently been used synonymously with “parent‐to‐infant attachment” [19–21]. The conceptual analysis of mother‐infant bonding by Altaweli and Roberts [20] included various expressions of attachment behaviors, which blurred the line between attachment and relationship‐building. Although the terms attachment and bonding are often used interchangeably in everyday language [19], inconsistencies are common in the research literature [21]. In the literature, the term “attachment,” proposed by Bowlby [22], refers to the relationship a child develops with a caregiver who aims to provide a secure foundation for exploring the world and, if it is necessary, protection, proximity, and a retreat for safety and comfort in conditions of stress and danger [22–25]. Prenatal attachment, on the other hand, refers to the emotional bond that develops between a pregnant woman and her fetus [26]. Müller and Mercer [27] defined prenatal attachment as a unique, emotional relationship that develops between the mother and fetus. This study adopts the prenatal attachment framework. A woman’s mental health during the prenatal period is related to her attachment to her unborn baby. A pregnant woman’s awareness of her own emotional tendencies is important for prenatal attachment. This attachment helps activate the mother’s care system and protect the fetus by preventing losses [28, 29]. Positive prenatal attachment can help pregnant women have a more positive birthing experience. On the other hand, low levels of prenatal attachment are negatively associated with the birthing experience in terms of labor duration, oxytocin use, and painkiller use (Smorti et al., 2020). Studies have shown that the perception of a traumatic birth is negatively related to prenatal attachment [13, 15].

Given the importance of maternal mental health for both mother’s and baby’s health, preventive and therapeutic support provided during pregnancy plays a critical role. In summary, studies have shown that depression, anxiety, and stress during pregnancy are significantly associated with a woman’s perception of childbirth as traumatic [10–13], and that the perception of traumatic childbirth is negatively related to prenatal attachment [13, 15]. Additionally, it has been reported that the prenatal mental health of pregnant women is related to their attachment to their unborn baby [28, 29]. Based on these findings, the present study aims to examine the relationships between traumatic childbirth perception and prenatal attachment, stress, anxiety, and depression among pregnant women.

Traumatic childbirth perception was considered the primary outcome variable, while prenatal attachment, stress, anxiety, and depression were treated as explanatory variables. No causal relationships were assumed due to the cross‐sectional design of the study. Based on the literature, the following hypotheses were formulated:

H1: Traumatic childbirth perception is positively associated with stress, anxiety, and depression.

H2: Traumatic childbirth perception is negatively associated with prenatal attachment.

H3: Prenatal attachment is negatively associated with stress, anxiety, and depression.

## 2. Method

### 2.1. Research Type

This study is designed as a descriptive and cross‐sectional study.

### 2.2. Study Population and Sample

The study’s population consisted of pregnant women visiting the obstetrics and gynecology outpatient clinic of a hospital located in the Central Black Sea region of Türkiye. A power analysis was performed using the G‐Power 3.1.9.7 program to calculate the sample size. With a margin of error (α = 0.05), 95% power, and an effect size of 0.20, the sample size was found to be 262. Considering the possibility of data loss during data collection, the study was completed with 304 pregnant women.

### 2.3. Inclusion Criteria

Applicants must be 18 years of age or older, have completed at least primary school education, be living with their spouse, be past the 20th week of pregnancy, be pregnant with a single child, not have a diagnosis of a psychiatric disorder, not have chronic diseases (such as hypertension, diabetes, and hyperthyroidism), not have experienced fetal problems or anomalies in the current pregnancy, not have a history of abnormal fetuses, births, or surviving children in previous pregnancies, or not be undergoing infertility treatment.

### 2.4. Exclusion Criteria

Women with chronic diseases (such as hypertension, diabetes, and hyperthyroidism), those with a high‐risk pregnancy, those experiencing fetal problems or anomalies in their current pregnancy, those with a history of abnormal fetuses, births, or surviving children in previous pregnancies, or those undergoing infertility treatment were excluded from the study. Additionally, women with clinically diagnosed psychiatric disorders were excluded because their anxiety, depression, and stress levels could be pathological, potentially acting as a confounding factor affecting the relationship between the variables studied.

### 2.5. Variables of the Study

The dependent variable of the study was traumatic childbirth perception. The independent variables included prenatal attachment, anxiety, stress, and depression. In addition, selected sociodemographic and obstetric characteristics, including age, gestational week, parity, and education level, were included as control variables.

### 2.6. Data Collection Method and Duration

Pregnant women gave their written and verbal consent. Data were collected between June 1, 2024, and June 5, 2025, using face‐to‐face interview techniques. The women themselves completed the questionnaire and scales in a quiet room in the hospital. Each woman completed the form in ~15 to 20 min.

### 2.7. Data Collection Tools

Data were collected using the Personal Characteristics Form, the Prenatal Attachment Inventory (PAI) [30], the Depression, Anxiety and Stress Scale (DASS‐21) [31], and the Traumatic Childbirth Perception Scale (TCPS) [32].

#### 2.7.1. Personal Information Form

This form also includes questions about participants’ age, height, weight, education level, occupation, family type, income level, duration of marriage, number of pregnancies, age at first pregnancy, gestational age, satisfaction with the baby’s gender, whether the pregnancy was planned, previous delivery method, assessment level of social support received during pregnancy, and assessment level of relationships with their spouse and mother.

#### 2.7.2. Depression, Anxiety, Stress Scale (DASS 21)

DASS‐21, which is the short form of the DASS 21, consisting of 42 items developed by Lovibond and Lovibond, was developed by Brown in 1997 [33, 34]. The DASS‐21 is a 4‐point Likert scale with its Turkish validity and reliability established by Yılmaz et al. [31]. The scale consists of 3 sub‐dimensions: depression, anxiety, and stress. There are 7 questions to measure each sub‐dimension. A minimum score of 0 and a maximum score of 21 can be obtained from each sub‐dimension. The scale is scored as follows: 0 “doesn’t apply to me at all,” 1 “applies to me a little,” 2 “usually applies to me,” and 3 “applies to me completely.” In the scoring of the scale, depression 0–4 points, anxiety 0–3 points, stress 0–7 points indicate a normal level; depression 5–6, anxiety 4–5 points, stress 8–9 points indicate a mild level; depression 7–10, anxiety 5–7 points, stress 10–12 points indicate a moderate level; depression 11–13, anxiety 8–9 points, stress 13–16 points indicate an advanced level; depression 14 and above points, anxiety 10 and above points, stress 17 and above points indicate a very advanced level problem [31]. Yılmaz et al. [31] reported that the Cronbach’s *α* value of the scale ranged from 0.755 to 0.822. In this study, Cronbach’s *α* was 0.808 for stress, 0.814 for depression, and 0.849 for anxiety.

#### 2.7.3. TCPS

The TCPS, developed by Yalnız et al. [32], is used to determine women’s perceptions of traumatic childbirth. The scale comprises 13 items, each scored from 0 (positive view) to 10 (negative view). The minimum possible score is 0, and the maximum is 130. The degree to which childbirth is perceived as traumatic is grouped as follows: 0–26 (very low), 27–52 (low), 53–78 (medium), 79–104 (high), and 105–130 points (very high). The scale has a single‐factor structure and does not contain reverse‐scoring items. As the score on the scale decreases, the degree to which childbirth is perceived as traumatic also decreases. The Cronbach α value, which was determined as 0.89 in the study by Yalnız et al. [32], was calculated as 0.92 in this study.

#### 2.7.4. The PAI

The scale was developed by Müller and Mercer [27] to assess the level of attachment, feelings, thoughts, and emotional states of pregnant women to their fetus. The Turkish adaptation, made by Yılmaz and Beji [30], is a four‐point Likert scale consisting of 21 items. Each item is scored on a scale of 1 to 4, where 1 means “never,” 2 means “sometimes,” 3 means “often,” and 4 means “always.” The minimum possible score on the scale is 21, and the maximum possible score is 84. There is no cutoff point. An increase in the scale score indicates an increase in the prenatal attachment level. The Cronbach *α* value, found to be 0.84 in the study by Yılmaz and Beji [30], was found to be 0.82 in this study.

### 2.8. Ethical Aspects of the Study

Permission for the scales used in the study was obtained from the authors via email. The study was approved by the Hitit University Non‐Interventional Clinical Research Ethics Committee (No. 2024/04, dated 07.02.2024). Permission for the study was obtained from the hospital. The women gave their consent, indicating that they were volunteers.

### 2.9. Data Analysis

All statistical analyses were performed using a statistical software package (SPSS 27.0). First, the completeness and accuracy of the data were checked. Descriptive statistics were calculated to summarize the characteristics of the participants and study variables. Continuous variables were presented as mean and standard deviation, while categorical variables were expressed as frequencies and percentages. The internal consistency reliability of the scales used in the study was evaluated using Cronbach’s alpha coefficients. To identify the predictors of traumatic childbirth perception, multiple linear regression analysis was performed. Traumatic childbirth perception was entered as the dependent variable, while prenatal attachment, depression, anxiety, and stress scores were included as independent variables. The regression results were reported using unstandardized coefficients (*B*), standard errors, standardized coefficients (*β*), *t* values, and *p* values. The overall fit of the model was evaluated using the coefficient of determination (*R* ^2^) and adjusted *R* ^2^. Prior to the regression analysis, the assumptions of linear regression, including linearity, normality, multicollinearity, and independence of errors, were examined. The level of statistical significance was set at *p*  < 0.05.

## 3. Results

The mean age of the participants in the study was 29.64 ± 5.73, the mean duration of marriage was 6.27 ± 5.24 years, the mean age at first pregnancy (i.e., the age at which the woman became pregnant for the first time in her life) was 25.08 ± 4.25, the mean number of pregnancies was 1.90 ± 1.04, and the mean gestational age was 31.77 ± 7.07 weeks. 40.1% of the women were university graduates, 55.9% were unemployed, 87.8% had a nuclear family type, and 63.2% had a middle‐income level. Furthermore, 97% of the participants reported being satisfied with the gender of their fetus, 75% reported that their pregnancy was planned, and 39.8% reported having had a vaginal birth before. Additionally, 71.7% of the pregnant women reported receiving good social support during pregnancy, 87.5% reported having a good relationship with their husbands, and 82.9% reported having a good relationship with their mothers (Table 1).

**Table 1:** Distribution of pregnant women according to sociodemographic and some characteristics.

Sociodemographic, obstetric, and social characteristics		*n*	%
Education level	Literate	2	0.7
	Primary school	22	7.2
	Middle school	41	13.5
	High school	117	38.5
	University	122	40.1
Occupation	Unemployed	170	55.9
	Civil servant	80	26.3
	Worker	25	8.2
	Self‐employed	29	9.5
Family type	Nuclear family	267	87.8
	Extended family	37	12.2
Income level	Low	9	3.0
	Middle	192	63.2
	High	103	33.9
Satisfaction with baby’s gender	Satisfied	295	97.0
	Dissatisfied	9	3.0
Whether pregnancy is planned	Planned	228	75.0
	Unplanned	76	25.0
Previous delivery method	Non‐birthing	121	39.8
	Vaginal	63	20.7
	Cesarean section	104	34.2
	Vaginal and cesarean section	16	5.3
Level of assessment of social support received during pregnancy	Good	218	71.7
	Moderate	79	26.0
	Poor	7	2.3
Level of assessment of relationship with spouse	Good	266	87.5
	Moderate	36	11.8
	Poor	2	0.7
Level of assessment of relationship with mother	Good	252	82.9
	Moderate	37	12.2
	Poor	15	4.9

		**X̄**	**SD**

Continuous variables	Age	29.64	5.73
	Weight	73.91	12.80
	Height	162.88	10.46
	Duration of marriage	6.27	5.24
	Number of pregnancies	1.90	1.04
	Age at first pregnancy	25.08	4.25
	Gestational age (weeks)	31.77	7.07

*Note:* %, percentage; $\overset{―}{X}$, mean.

Abbreviations: *n*, number; SD, standard deviation.

**Figure 1:**
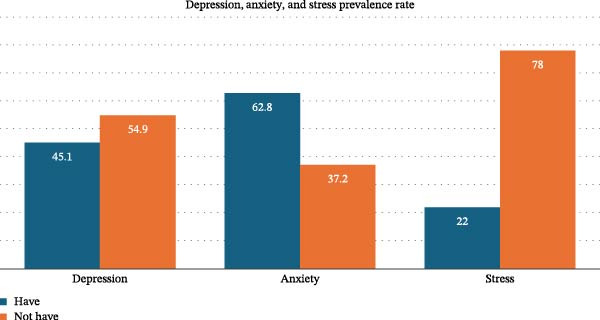
Distribution of depression, anxiety, and stress rates in pregnant women.

The study found that 62.8% of women experienced anxiety (cutoff score >7), 22% experienced stress (cutoff score >14), and 45.1% experienced depression (cutoff score >9). (Figure 1). It was determined that pregnant women had PAI scores of 40.53 ± 7.97 and TCPS scores of 71.64 ± 28.02 (moderate).

Pearson correlation analysis was conducted to examine the relationships between prenatal attachment, traumatic childbirth perception, and psychological variables (depression, anxiety, and stress). The results indicated that prenatal attachment was significantly and negatively correlated with depression (*r* = −0.227, *p*  < 0.001), anxiety (*r* = −0.232, *p*  < 0.001), and stress (*r* = −0.239, *p*  < 0.001). These findings suggest that higher levels of depression, anxiety, and stress were associated with lower levels of prenatal attachment. In contrast, traumatic childbirth perception was significantly and positively correlated with depression (*r* = 0.337, *p*  < 0.001), anxiety (*r* = 0.302, *p*  < 0.001), and stress (*r* = 0.277, *p*  < 0.001), indicating that higher levels of psychological distress were associated with higher levels of traumatic childbirth perception. However, no significant relationship was found between prenatal attachment and traumatic childbirth perception (*r* = 0.050, *p* = 0.388). Additionally, strong positive correlations were observed among the psychological variables themselves. Depression showed a strong positive correlation with anxiety (*r* = 0.850, *p*  < 0.001) and stress (*r* = 0.861, *p*  < 0.001), while anxiety was also strongly correlated with stress (*r* = 0.865, *p*  < 0.001) (Table 2).

**Table 2:** Relationship between prenatal attachment inventory, traumatic childbirth perception scale, depression, anxiety, and stress scores in pregnant women.

Scales and subscales	PAI	TCPS	Depression	Anxiety	Stress
PAI					
*r*	1	—	—	—	—
*p*	—	—	—	—	—
TCPS					
*r*	0.050	1	—	—	—
* p*	0.388	—	—	—	—
Depression					
*r*	−0.227	0.337	1	—	—
*p*	**0.000** ^∗^	**0.000** ^∗^	—	—	—
Anxiety					
*r*	−0.232	0.302	0.850	1	—
*p*	**0.000** ^∗^	**0.000** ^∗^	**0.000** ^∗^	—	—
Stress					
*r*	−0.239	0.277	0.861	0.865	1
*p*	**0.000** ^∗^	**0.000** ^∗^	**0.000** ^∗^	**0.000** ^∗^	—

*Note: r*: Pearson correlation coefficient. Statistically significant values are shown in bold.

Abbreviations: PAI, Prenatal Attachment Inventory; TCPS, Traumatic Childbirth Perception Scale.

^∗^ *p* < 0.05.

According to regression analysis, depression and prenatal attachment were determined to be predictors of traumatic childbirth perception in pregnant women (*R =* 0.370, *R* ^2^ = 0.137, *Adj. R* ^2^ = 0.072, *p*  < 0.05). Accordingly, the model explains 13.7% of the total variance related to the perception of traumatic birth. The standardized regression coefficient (*β*) indicates that the relative significance of the predictor variables for the perception of traumatic birth is highest for depression (*β* = 0.348) and prenatal attachment (*β* = 0.132). When the significance of the regression coefficients was examined, depression (*p* = 0.003) and prenatal attachment (*p* = 0.018) were identified as significant predictors of the perception of traumatic childbirth (Table 3).

**Table 3:** Predictors of traumatic childbirth perception in pregnant women.

Model	Unstandardized coefficients		Standardized coefficients	*t*	*p*‐Value
	*B*	Std. error	Beta		
Constant	24.155	10.269	—	2.352	0.019
PAI	0.464	0.196	0.132	2.371	**0.018**
Depression	2.382	0.801	0.348	2.973	**0.003**
Anxiety	0.724	0.746	0.115	0.971	0.333
Stress	−0.660	0.886	−0.092	−0.745	0.457

*Note: R* = 0.370, *R* ^2^ = 0.137, Adj. *R* ^2^ = 0.072, *p*  < 0.05. Statistically significant values are shown in bold.

Abbreviation: PAI, Prenatal Attachment Inventory.

## 4. Discussion

This study examined the relationship between the perception of traumatic childbirth and prenatal attachment, anxiety, stress, and depression in pregnant women.

Physical changes during pregnancy, maternal and infant health, and parental roles create challenges for women’s mental health [35]. Depression, stress, and anxiety are among the most common mental health problems during pregnancy [36]. In this study, based on the threshold values of the anxiety, stress, and depression subscales, it was determined that 45.1% of pregnant women experienced depression, 22% experienced stress, and 62.8% experienced anxiety (Figure 1). Another study conducted in Turkey reported that 37.2% of pregnant women experienced depression, 40.9% experienced stress, and 52.8% experienced anxiety symptoms [37]. A study conducted on pregnant women in China revealed that 19.4% experienced depressive symptoms, 26.6% experienced anxiety, and 92.3% experienced stress symptoms [38]. Compared to Kiyak’s [37] study, which had a similar sample size and used the same measurement tool, participants in this study were found to have higher levels of depression and anxiety, and lower levels of stress. This may be related to factors such as differences in sample characteristics (gestational age, number of births, or pregnancy risk status, etc.), regional socioeconomic conditions, access to prenatal care, and levels of social support. The particularly high prevalence of anxiety may be associated with uncertainty during pregnancy, concerns about childbirth, and perceived risks to maternal and infant health. The particularly high prevalence of anxiety may be associated with uncertainty during pregnancy, concerns about childbirth, and perceived risks to maternal and infant health. Furthermore, when compared with the findings of the study conducted in China by Meng et al. [38], the observed differences indicate that cultural and contextual factors may influence how psychological distress is experienced and reported during pregnancy. These findings highlight the importance of considering contextual factors and support the integration of routine mental health screening into prenatal care.

In this study, participants’ TCPS scores (71.64 ± 28.02) were found to be at a moderate level. Similarly, other studies have also reported moderate average TCPS scores [12, 15]. In contrast, Görgün and Taşğın [39] reported higher average TCPS scores (78.82 ± 26.40). This suggests that the levels of traumatic birth perception may vary in different samples. The TCPS scores observed in the present study may be associated with the psychological characteristics of the sample, particularly levels of anxiety and depression. Psychological distress during pregnancy may increase the tendency to perceive childbirth as threatening or uncontrollable, thereby influencing traumatic childbirth perceptions. In addition, differences between studies may be related to contextual factors such as variations in healthcare settings, perceived quality of care, and levels of social support. These findings highlight the importance of early evaluation of childbirth‐related perceptions and the provision of supportive interventions during pregnancy.

In this study, the mean PAI score was 40.53 ± 7.97. While some studies [40, 41] found similar PAI scores, others [29, 42] found higher scores. This suggests variability between different samples. The relatively low prenatal attachment levels observed in this study may be related to participants’ psychological characteristics. Specifically, participants’ levels of anxiety, depression, and perceived traumatic birth may have negatively impacted the mother’s emotional bond with the fetus. In addition, differences between studies may be explained by contextual and methodological factors, such as variations in sample characteristics, social support, cultural expectations regarding motherhood, and the timing of assessment.

This study found that prenatal attachment was significantly negatively correlated with depression, stress, and anxiety (Table 2). Consistent with this study, a systematic review by Rollè et al. [43] reported a negative association between prenatal depressive symptoms and prenatal attachment. Similarly, Medina et al. [44] determined that high levels of depression hurt prenatal attachment. Şanlı and Akbağ [41] found a negative association between prenatal attachment and pregnancy stress in their study. Dayton et al. [45] reported that maternal psychological distress is related to mother–infant attachment. Camarneiro et al. [46] reported that depression and anxiety significantly contributed to explaining maternal prenatal attachment. Additionally, Choe et al. [47], studying high‐risk pregnancies, and Maghalian et al. [48], examining unplanned pregnancies, found that higher maternal anxiety and depression were associated with lower prenatal attachment. These consistent findings across diverse samples suggest that maternal mental health is a robust determinant of prenatal attachment. Contextual factors in the present study—such as cultural expectations about pregnancy, perceived social support, and concerns about maternal and fetal health—may have contributed to the observed correlations. Methodological aspects, including self‐report measures and recruitment from urban obstetric clinics, could also influence these associations. Overall, these results underscore the importance of early psychological screening and supportive interventions to foster maternal‐fetal bonding during pregnancy.

Women’s negative perceptions about childbirth can lead to depression and anxiety, negatively impacting their psychosocial health [12]. This study determined a significantly positive correlation between anxiety, depression, and stress scores and the perception of traumatic childbirth (Table 2). One study reported that pregnant women with high health anxiety had higher traumatic birth perception scores [11]. In another similar study, it was found that pregnant participants with high traumatic birth perception scores also had higher depression and anxiety scores [12]. In the study conducted by Ma et al. [49], it was found that as pregnancy stress increases, psychological birth traumas also increase. These associations may be influenced by contextual factors, such as cultural expectations regarding childbirth, perceived social support, and concerns about maternal and fetal health. Methodological factors, including self‐report measures and recruitment from urban obstetric clinics, could also affect the observed relationships. Overall, these findings suggest that psychosocial interventions targeting anxiety, stress, and depressive symptoms may reduce the risk of perceiving childbirth as traumatic, highlighting the need for early screening and supportive care in prenatal settings.

This study found that depression and prenatal attachment were significant predictors of perceived traumatic childbirth, with the regression model explaining a relatively low proportion of the variance (*R* ^2^ = 0.137). Similar findings have been reported in previous studies, indicating that depressive symptoms are associated with higher perceived traumatic childbirth [12, 50]. The finding that prenatal attachment predicts traumatic childbirth perception suggests that emotional processes during pregnancy may influence how women interpret and anticipate the childbirth experience. However, rather than indicating a direct causal relationship, this association may reflect broader psychological and contextual dynamics, such as heightened emotional involvement, perceived vulnerability, or concerns about maternal and fetal well‐being. In contrast, some studies have approached this relationship from the opposite direction. For example, Şahin and Erbil [15] reported that traumatic childbirth perception predicts mother–infant attachment, whereas Yalniz Dilcen et al. [29] found that traumatic childbirth perception was not a significant indicator of prenatal attachment. These inconsistencies may be related to differences in study design, timing of measurement, and sample characteristics. The relatively low explanatory power of the model indicates that a substantial proportion of variance remains unexplained, suggesting the influence of additional factors such as social support, childbirth preparation, previous experiences, and characteristics of the healthcare system. From a clinical perspective, these findings highlight the importance of integrating routine psychological assessment into prenatal care. In particular, midwives and nurses should evaluate both maternal mental health and prenatal attachment and provide appropriate psychosocial support to improve childbirth perceptions. Future research should adopt longitudinal designs and include broader psychosocial and contextual variables to better explain traumatic childbirth perception.

### 4.1. Limitations of the Study

One limitation of this study is that the measurement tools were based on self‐reported data, which may be subject to response bias. In addition, the findings are limited to pregnant women in a single region and may not be generalizable to other populations. The data were collected within a specific time frame, which may not reflect changes over time.

Another important limitation is the cross‐sectional design of the study, which prevents drawing causal inferences about the relationships between prenatal attachment, mental health variables, and the perception of traumatic childbirth.

Future studies should use longitudinal designs more effectively better to understand the temporal and causal relationships between these variables. In addition, including other psychosocial factors such as social support, coping strategies, and obstetric characteristics may provide a more comprehensive understanding of traumatic childbirth perception. Intervention‐based studies are also recommended to evaluate the effectiveness of programs aimed at improving maternal mental health and prenatal attachment.

## 5. Conclusion

This study revealed the critical role of psychological well‐being during pregnancy on prenatal bonding and perception of birth. The study findings highlight that anxiety, stress, and depression experienced during pregnancy are significantly associated with prenatal attachment and perceptions of traumatic birth. However, given the cross‐sectional design, causal inferences cannot be made, and these relationships should be interpreted cautiously.

The findings underscore the potential importance of maternal psychological well‐being in shaping both prenatal attachment and perceptions of childbirth. From a clinical practice perspective, it is recommended that nurses integrate standard mental health screening protocols into prenatal care, going beyond mere physical monitoring. These screenings can help identify women at risk and support early intervention. Cognitive interventions addressing perceptions of traumatic childbirth and support groups to strengthen prenatal attachment should be structured, particularly for pregnant women in at‐risk groups.

Future research should utilize longitudinal and intervention‐based designs to better understand causal relationships and assess the effectiveness of psychosocial support programs. Additionally, incorporating a broader range of psychosocial and contextual variables, such as partner relationships, perceived social support, and previous birth experiences, may provide a more comprehensive understanding of the factors influencing traumatic childbirth perceptions.

## Author Contributions

**Hüsne Yücesoy:** conceptualization, methodology, writing – original draft, investigation, data curation, formal analysis, writing – review & editing. **Fatma Yildirim:** conceptualization, methodology, writing – original draft, data curation, formal analysis. **Mevlüde Alpaslan Arar:** conceptualization, writing – original draft, data curation. **Nülüfer Erbil:** conceptualization, formal analysis, writing – review & editing.

## Funding

No funding was received for this manuscript.

## Disclosure

All coauthors revised and agreed to publish the final version of the manuscript.

## Conflicts of Interest

The authors declare no conflicts of interest.

## Data Availability

The data that support the findings of this study are available from the corresponding author upon reasonable request.
